# Waterpipe Use and Its Cardiovascular Effects: A Systematic Review and Meta-Analysis of Case-Control, Cross-Sectional, and Non-Randomized Studies

**DOI:** 10.7759/cureus.34802

**Published:** 2023-02-09

**Authors:** Kamran Mahfooz, Advait M Vasavada, Arpit Joshi, Srikrishnan Pichuthirumalai, Rupesh Andani, Arush Rajotia, Aakash Hans, Bilvesh Mandalia, Neeraj Dayama, Zara Younas, Nosheen Hafeez, Niharika Bheemisetty, Yash Patel, Hemalatha Tumkur Ranganathan, Ashok Sodala

**Affiliations:** 1 Internal Medicine, Lincoln Medical Center, New York, USA; 2 Internal Medicine, California Institute of Behavioral Neurosciences & Psychology, Fairfield, USA; 3 Medicine, Shri M. P. Shah Government Medical College, Jamnagar, IND; 4 Medicine, B. J. (Byramjee Jeejeebhoy) Medical, Ahmedabad, IND; 5 Internal Medicine, Sakra World Hospital, Bengaluru, IND; 6 Internal Medicine, Jeevandhara Hospital, Jamnagar, IND; 7 Cardiology, Mayo Clinic, Rochester, USA; 8 Internal Medicine, Henry Ford Health System, Detroit, USA; 9 House Officer, Lokmanya Tilak Municipal General Hospital and Medical College, Sion Mumbai, Mumbai, IND; 10 Internal Medicine, Texas Tech University Health Sciences Center, Lubbock, USA; 11 Medicine, King Edward Medical University, Lahore, PAK; 12 Pediatrics, California Institute of Behavioral Neurosciences & Psychology, Fairfield, USA; 13 Medicine, Gujarat Cancer Society Medical College, Ahmedabad, IND; 14 Medicine, Mandya Institute of Medical Science, Mandya, IND

**Keywords:** cardiovascular disease, non-randomized studies, heated tobacco products, waterpipe, tobacco use disorder

## Abstract

Approximately 100 million people globally smoke cigarettes, making it a significant and quickly spreading global tobacco epidemic. Substance use disorders are frequently evaluated by non-randomized studies. Tobacco use and its impacts on the cardiovascular system were the subjects of a comprehensive search across five electronic databases: Cochrane, MEDLINE, Scopus, Embase, and PubMed. The findings demonstrated that waterpipe smokers in comparison to non-smokers have immediate elevations in heart rate and blood pressure, lower levels of high-density lipoprotein, higher levels of low-density lipoprotein, higher levels of triglycerides, higher levels of fasting blood glucose, and a higher heart rate. Users of waterpipes and cigarettes had similar average heart rates, blood pressure, and lipid levels, with the exception that waterpipe smokers had greater total cholesterol. Smoking a waterpipe has significant negative effects on the cardiovascular system comparable to cigarette smoking, and non-randomized studies proved to yield substantial evidence related to its cardiovascular effects. Such study designs can be used to evaluate substance use and its cardiovascular impact.

## Introduction and background

The main cause of mortality worldwide in 2015 was cardiovascular disease (CVD), which was responsible for 17.9 million deaths worldwide [1]. According to estimates, ventricular tachyarrhythmias account for roughly 80% of all sudden cardiac deaths, which account for around 40-50% of all cardiovascular (CV) deaths [2]. For instance, each year, between 250,000 and 310,000 sudden cardiac fatalities occur in the United States [3,4]. A cardiac arrhythmia is what causes sudden cardiac death, which is why the majority of cardiac arrests are fatal and usually occur without any prior warning signals [5-7]. The majority of sudden cardiac deaths occur in the general population and people without established coronary heart disease [8,9], despite the fact that preventive efforts have primarily focused on using cardioverter-defibrillators in the highest-risk groups, such as patients with advanced cardiomyopathy and reduced left ventricular ejection fraction [10]. Therefore, it stands to reason that population-wide primary preventive initiatives would be a more effective strategy to reduce sudden cardiac fatalities. Age, obesity, diabetes, inactivity, dietary variables, hypertension, high serum cholesterol, a high resting heart rate (HR), and a family history of sudden cardiac death are all recognized or suspected risk factors for sudden cardiac death [11,12]. A number of cohort studies have also found that smoking is strongly associated with an increased risk of sudden cardiac death; however, the intensity of the relationships observed has ranged from a 50% increase in risk to a 5.5-fold increase in risk [13-16]. The length of follow-up, geographic location, the definition of the reference group, discrepancies in sample numbers between studies, and/or chance fluctuation may all contribute to variations in effect sizes.

Waterpipe use has grown into a significant and quickly spreading global tobacco problem. The stylish feature of WPS (waterpipe smoking) and several other factors, particularly flavored smoke, have made it difficult to recognize the detrimental effects of WPS [17]. Furthermore, the water appears to remove the majority of hazardous chemicals from the smoke. The assumption made by users that the smoke is "filtered" by the water is false because a single WPS session lasts 30 to 90 minutes of nonstop smoking [18,19]. This prolonged period of time results in a large volume of smoke that contains up to 80 times more toxicants than those found in the smoke of a single cigarette and is carried through the water in the bubbles. It could be detrimental similar to cigarette smoking (CS). WPS may have negative CV effects, which have been observed in a number of dispersed research studies with varying findings based on various estimation methodologies. The total clinical impact of WPS on the CV system is not yet known. A few available studies on this topic had weak validity since they did not synthesize their data or were especially not concerned with CV outcomes [18,20-22]. To better understand the connection between WPS and CVD risk, we investigate the clinical CV effects of WPS statistically and qualitatively, compare them with those of tobacco smoking, and combine all relevant data from non-randomized studies.

## Review

Methods

Study Design and Data Sources

In accordance with the guidelines and concepts outlined by the PRISMA (Preferred Reporting Items for Systematic Reviews and Meta-Analyses) framework, the current article is a systematic review and meta-analysis of case-control, cross-sectional, and non-randomized controlled trials [23]. We sought to identify and review the quality of literature pertaining to the topic in such studies. Five medical databases were thoroughly examined for admissible primary research pertinent to the topic at hand. To find English-language literature examining tobacco use and its effects on the CV system, searches were conducted in the following databases: Cochrane, Embase, MEDLINE, PubMed, and Scopus.

Search Strategy

This article’s keywords and key concepts were the focus of a detailed search strategy. The search procedure also included the Boolean expression, which mainly consisted of “AND” and “OR.” The terms “Tobacco smoking,” OR “Cigarette smoking,” OR “Waterpipe smoking,” AND “CVD,” OR “cardiovascular effects,” OR “cardiovascular disease” were utilized to the fullest extent possible. The search was restricted to research studies that were published in English.

Eligibility Criteria

The following inclusion criteria were applied to narrow down the pool of source articles for careful selection for analysis in this study: original articles, non-randomized studies, and English-language articles published between 2010 and 2022 examine the relationship between tobacco use and the risk of CVD.

On the other hand, studies were rejected and not taken into account based on the following criteria: secondary sources, such as journals, newspapers, and other academic research, studies that discuss the impacts of cigarette smoking and CVD, and studies that look at cigarette usage without considering the effects of CVD. Case studies and other study kinds were also discarded, as was the publication of main papers in languages other than English language, to prevent information loss and distortion through translation.

Data Extraction Quality Assessment

Two independent reviewers were in charge of choosing and extracting data from research that met the inclusion requirements for the PICO (Population, Intervention, Comparison, Outcome) framework [24]. Details on the authors, study methods, participant characteristics, intervention, comparison, and key findings or outcomes were among the factors that these reviewers acquired. A second reviewer was consulted to help with the harmonization and extraction of pertinent data for data and statistical analysis. This was done to address issues with data extraction. The Cochrane risk-of-bias tool was also utilized to assess the caliber of the study. This method uses six criteria, including reporting, blinding, selection, binding, attrition, and other biases, to categorize studies as having a low, high, or unknown risk of bias [25].

Statistical Analysis

It was effectively used to ensure that data analysis was conducted in accordance with the needs, and the data were gathered using the Cochrane Review Manager Software (RevMan version 5.4). According to the Cochrane criteria, each study utilized in a meta-analysis must have a consistent design and use comparable metrics. The mean difference (MD) and poled odds ratio were selected as the effect measures, each with a 95% level of confidence. The I-square (I^2^) test was employed to determine heterogeneity, with an I^2^ threshold of >50% signifying high heterogeneity and below 50% signifying low heterogeneity. The threshold for statistical significance was set at p<0.05 [26].

Results

Search Results

**Figure 1:**
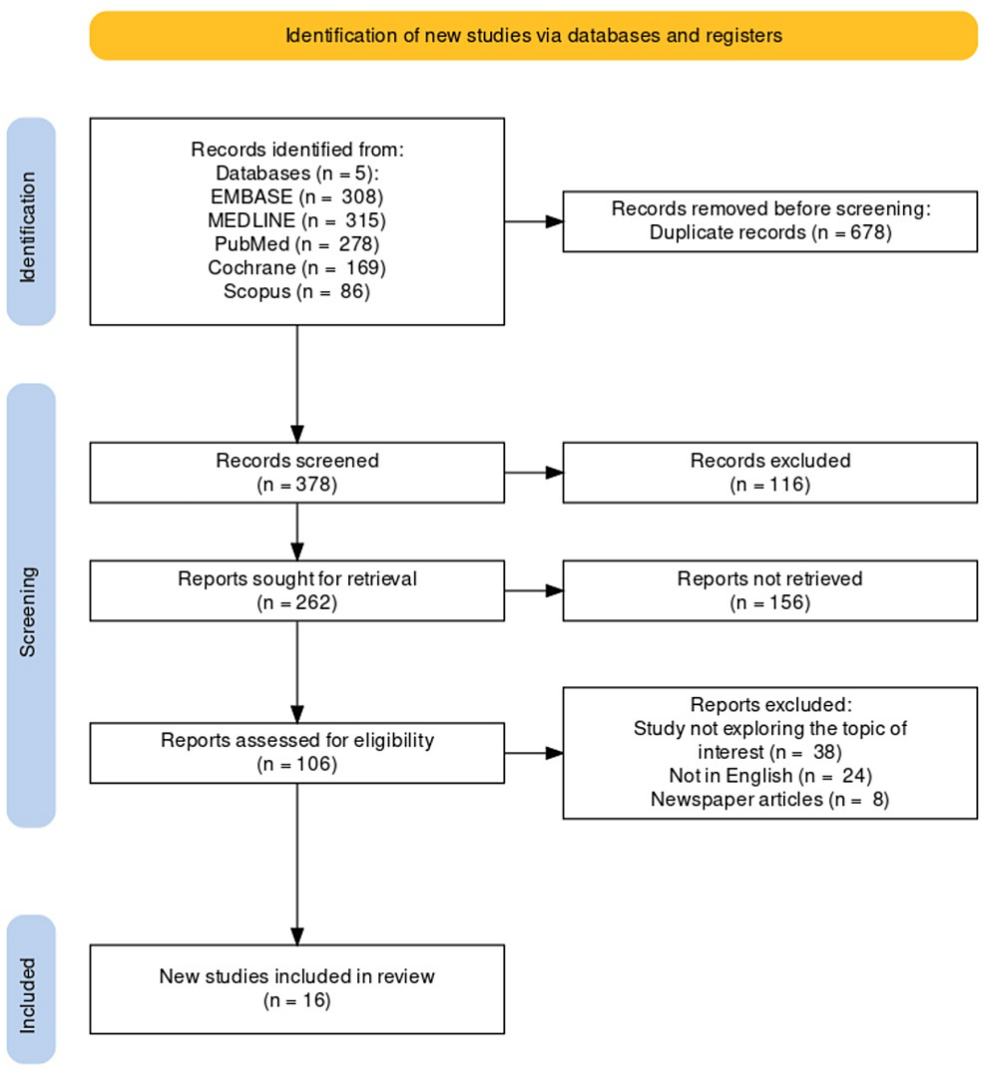
The search procedure depicted in the PRISMA flowchart was used to locate the 16 studies. PRISMA, Preferred Reporting Items for Systematic Reviews and Meta-Analyses; MEDLINE, Medical Literature Analysis and Retrieval System Online; EMBASE, Excerpta Medica Database

All 1,056 articles contained citations located in several databases besides searching through their reference lists. Only 16 of these non-randomized studies, all of which addressed the link between tobacco use and the risk of CVD, were found to meet the inclusion requirements and were pooled for analysis. The search strategy used to find the 16 articles pertinent to this inquiry is depicted in Figure 1. The number of studies not retrieved was 156 and, they were either paid access, no full text, or without valid website/journal links.

Study Characteristics

Table 1 describes the characteristics of the 16 included articles.

**Table 1:** Study characteristics ES, experimental studies; CSS, cross-sectional studies; CCS, case-control studies; NS, not specified; WPS, waterpipe smoking

Study	Study design	Participants			WPS		Intervention			Outcomes
		N	Male	Age, mean (years)	Total	Frequency	Pre-session abstinence	Smoking settings	Smoking duration (min)	Tobacco used
Blank et al. (2011) [27]	ES	37	29	20.5	37	≤5 cigarettes per month	Overnight	Laboratory	45	10 grams of tobacco
Alomari et al. (2014) [28]	ES	53	34	22.7	53	≥3 WPS per week	NS	Well-ventilated room	30	10 grams
Azar et al. (2016) [29]	ES	194	112	35.6	101	NS	12 hours	Restaurants	15	NS
Bentur et al. (2014) [30]	ES	62	33	24.9	47	NS	24 hours	Indoor	30	10 grams of moassal
Al-Amri et al. (2019) [31]	CCS	296	203	47.8	35	Daily	NS	NS	-	-
Chami et al. (2019) [32]	CCS	345	233	53.7	175	Daily	-	-	-	NS
Ghasemi et al. (2010) [33]	CCS	54	54	33.3	27	Daily	NS	-	-	Frequently moassal
Al Suwaidi et al. (2012) [34]	CSS	7930	6253	59.6	130	Regular	-	-	-	NS
Khan et al. (2020) [35]	CSS	73	41	39.8	12	Daily	-	-	-	NS
Platt et al. (2017) [36]	CSS	7705	5188	61.2	574	Regular	-	-	-	NS
Nelson et al. (2016) [37]	ES	28	20	27	28	12 times in the past year	72 hours	Laboratory	30	NS
Chwyeed (2018) [38]	CCS	75	75	30	20	NS	NS	-	-	NS
Diab et al. (2015) [39]	CCS	77	77	35.1	30	Daily	NS	-	-	NS
Rezk-Hanna and Benowitz (2019) [20]	ES	55	10	26	40	More than 12 times a year	Overnight	Laboratory	40	NS
Saffar Soflaei et al. (2018) [40]	CSS	9690	NS	35	1067	NS	-	-	NS	NS
Selim et al. (2013) [41]	CSS	70	63	28.7	30	Daily	NS	-	-	NS

Risk-of-Bias Evaluation

**Figure 2:**
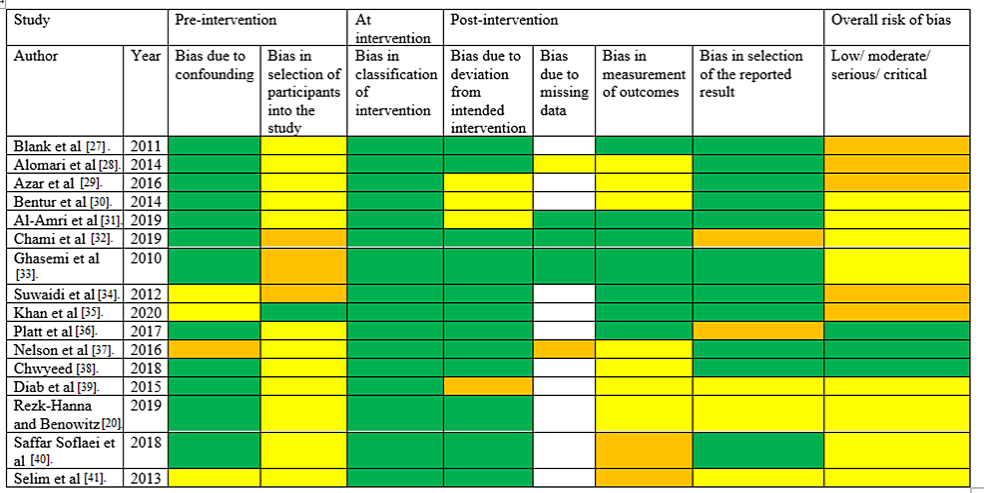
Risk-of-bias assessment, with green, yellow, orange, and white indicating low, moderate, serious, and critical risks, respectively. [20,27-41]

Two researchers independently evaluated the risk of bias using the ROBINS-I (Risk Of Bias In Non-Randomized Studies - of Interventions) instrument, as depicted in Figure 2, which the Cochrane Bias Methods Group recommends for evaluating the risk of bias in non-randomized interventions [42]. A third investigator was consulted in the event of differences.

Acute Effects of Tobacco Smoking

**Figure 3:**
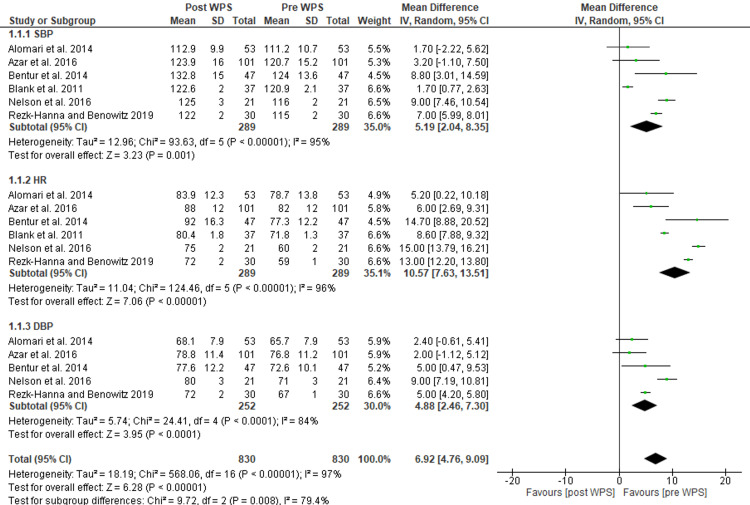
Plot demonstrating the acute effect of WPS. WPS, waterpipe smoking; HR, heart rate; SBP, systolic blood pressure; DBP, diastolic blood pressure [20,27-30,37]

A meta-analysis was conducted for each of the three measures, HR, systolic blood pressure (SBP), and diastolic blood pressure (DBP), to determine the acute impact of WPS on each. The findings demonstrated that a single WPS session resulted in acute increases in mean HR (MD: 10.57; 95% CI: 7.63 to 13.51; I^2^ = 96%), SBP (MD: 5.19; 95% CI: 2.04 to 8.35; I^2^ = 95%), and DBP (MD: 4.88; 95% CI: 2.46 to 7.30; I^2^ = 84%). The acute effect of WPS on these three hemodynamic measures remained significant even when statistical heterogeneity in the sensitivity analysis was eliminated (Figure 3).

Non-Acute Effects

**Figure 4:**
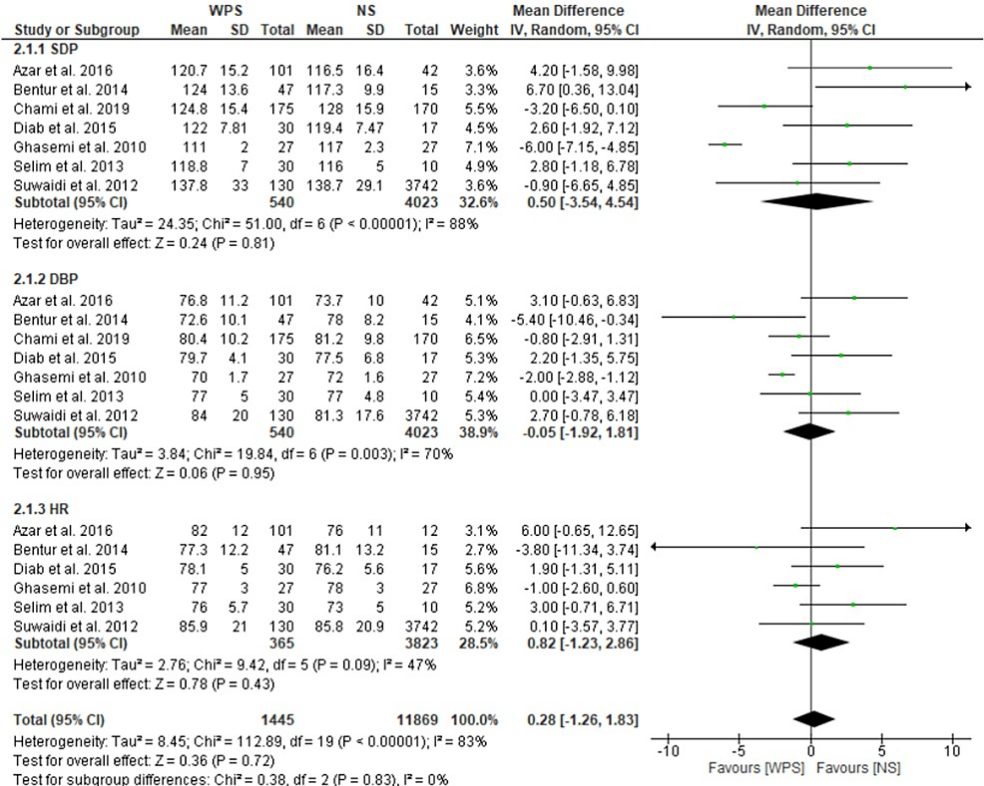
Forest plot showing a comparison of WPS and NS. WPS, waterpipe smoking; NS, non-smoking [29,30,32-34,39,41]

According to the findings, waterpipe smokers tend to have greater blood pressure (BP) than non-smokers, with a mean HR that is higher (MD: 0.82; 95% CI: -1.23 to 2.86; I^2^ = 47%). Once the statistical heterogeneity from the sensitivity studies was considered, there was no significant correlation between WPS and any of these hemodynamic measures. The overall pooled effect was insignificant (MD: 0.28; 95% CI: -1.26 to 1.83; I^2^ = 83%) (Figure 4).

Lipoproteins

**Figure 5:**
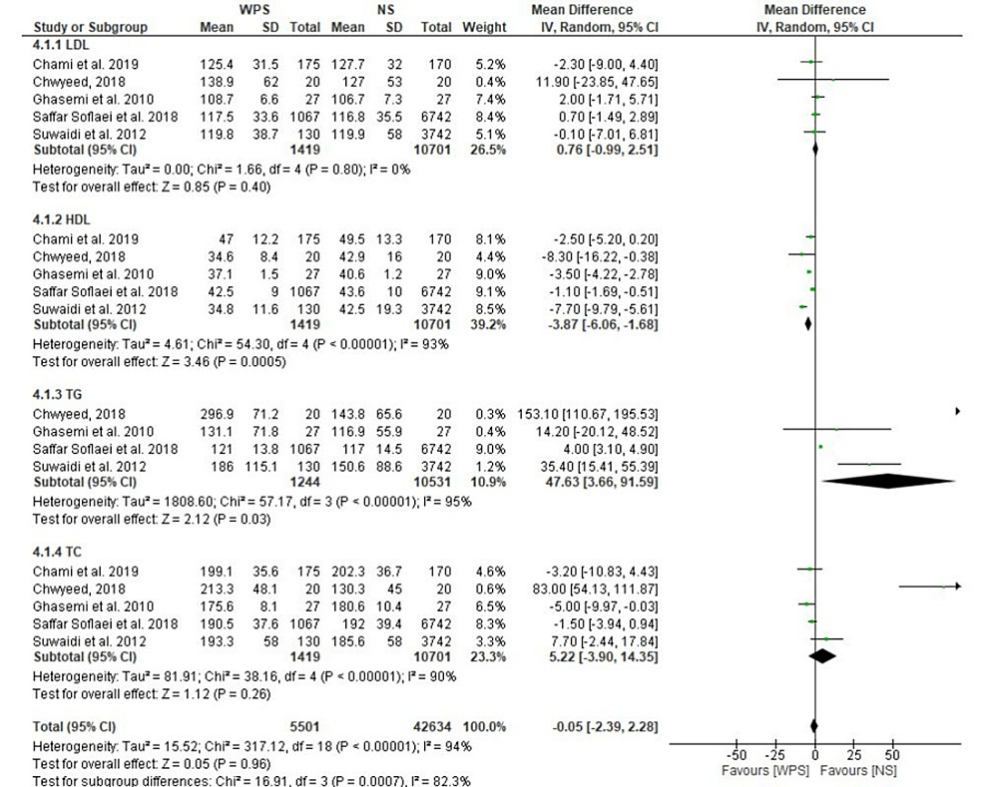
Forest plots showing individual and pooled mean differences in cholesterol blood values in WPS when compared to NS. WPS, waterpipe smoking; NS, non-smoking; TC, total cholesterol; LDL, low-density lipoprotein; HDL, high-density lipoprotein; TG, triglycerides [32-34,38,40]

By performing the meta-analysis for each of TC (total cholesterol), LDL (low-density lipoprotein), HDL (high-density lipoprotein) cholesterol, and TG (triglycerides), and having dyslipidemia, it was possible to determine whether WPS and serum lipid levels are correlated. The results showed that when compared to non-smokers, waterpipe smokers had lower mean HDL cholesterol (MD: -3.87; 95% CI: -6.06 to -1.68), higher mean TG (MD: 47.63; 95% CI: 3.66 to 91.59), and higher mean LDL cholesterol (MD: 0.76; 95% CI: -0.99 to 2.51; I^2^ = 0%). Even after statistical heterogeneity in the sensitivity analysis was eliminated, there was still a significant connection between WPS and higher TG levels and lower HDL cholesterol (Figure 5).

**Figure 6:**
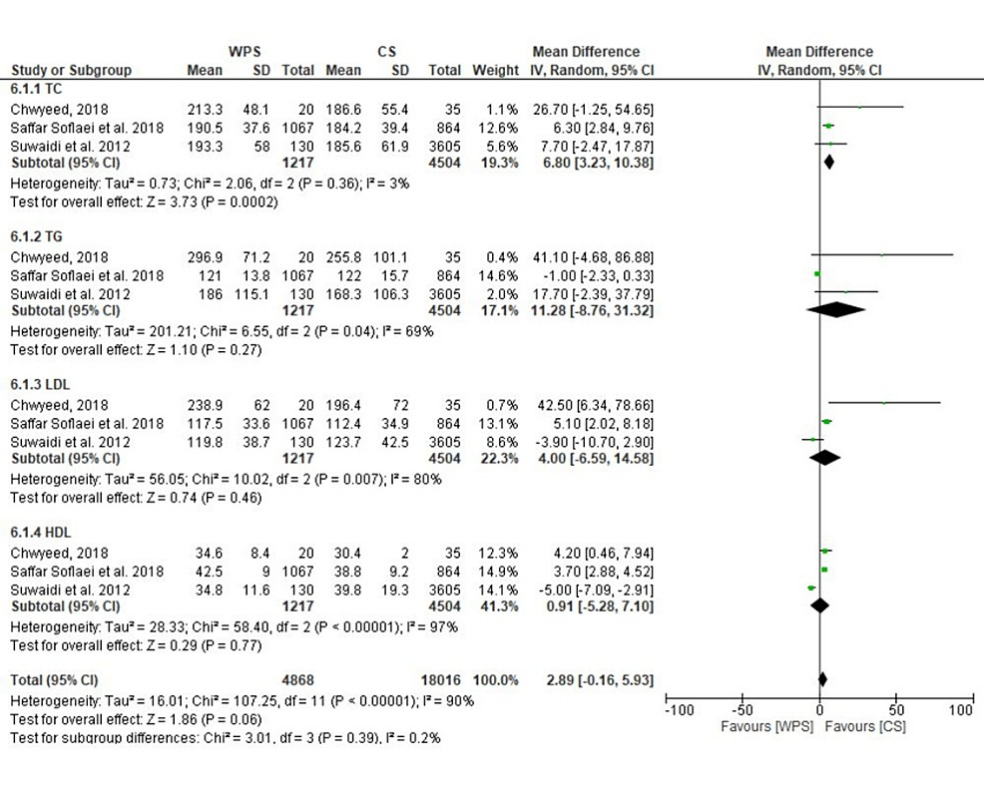
A forest plot demonstrating HDL, LDL, TC, and TG pooled mean differences in WPS versus CS. WPS, waterpipe smoking; CS, cigarette smoking; TC, total cholesterol; LDL, low-density lipoprotein; HDL, high-density lipoprotein; TG, triglycerides [34,38,40]

Data from three trials were combined, and the results showed that waterpipe users had greater mean TC than cigarette smokers (MD: 2.89; 95% CI: -0.16 to 5.93; I^2^ = 90%). No differences were discovered in terms of TG, dyslipidemia, LDL, HDL, or TG values. However, reducing the statistical heterogeneity in sensitivity analysis indicated a strong association between WPS and TG levels (Figure 6).

Cardiovascular Effects

**Figure 7:**
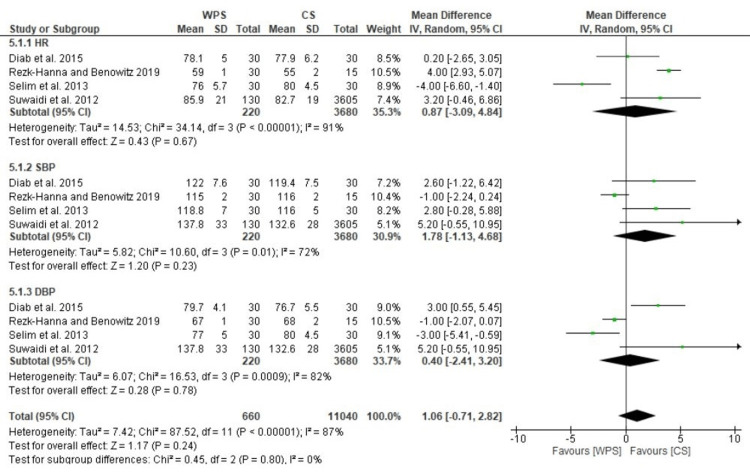
A plot showing a comparison of WPS and CS. WPS, waterpipe smoking; CS, cigarette smoking; HR, heart rate; SBP, systolic blood pressure; DBP, diastolic blood pressure [20,34,39,41]

A meta-analysis revealed no HR, SBP, or DBP changes between waterpipe and cigarette smokers. Sensitivity analyses revealed significantly higher mean HRs (MD: 0.87; 95% CI: -3.09 to 4.84; I^2^ = 91%), SBPs (MD: 1.78; 95% CI: -1.13 to 4.68; I^2^ = 72%), and DBPs (MD: 0.40; 95% CI: -2.41 to 3.20; I^2^ = 82%) after statistical heterogeneity was removed (Figure 7).

Discussion

Increased HR and BP, the two most common hemodynamic parameters used to evaluate the CV system, are known to have a deleterious impact on CV outcomes [43,44]. Our findings show that a single WPS session significantly raises HR, SBP, and DBP. This alone could increase the heart's need for oxygen, increase blood vessel shear stress, and occasionally trigger ACS (acute coronary syndrome), raising morbidity and death. It is reasonable to assume that the accumulation of these acute adverse effects will have a negative long-term impact on prognosis because waterpipe is typically consumed regularly several times a week. Our findings, however, indicate that while SBD and DBP tend to be higher among waterpipe smokers, they do not statistically differ from those who do not smoke in terms of HR from non-smokers. These results are somewhat unexpectedly different from those seen with WPS's acute effects, which could be partially explained by the studies' significant heterogeneity. Additionally, years of smoking and the frequency and length of WPS sessions were not controlled between trials, which could have an impact on the findings. The frequency of weekly waterpipe use was previously found to be significantly positively correlated with SBP, SBP, and HR [45]. The nicotine exposure that raises the sympathetic nervous system's activity and causes an increase in HR, myocardial contractility, and cardiac output may be partially responsible for the acute hemodynamic abnormalities identified in our findings [46]. Three trials contrasting flavor-matched tobacco with tobacco-free WPS have revealed such an effect [27,47,48]. However, regardless of nicotine concentration, an immediate cardiac autonomic dysregulation was seen after a WPS session.

Additionally, due to the creation of carboxy-hemoglobin (CO-Hb), the high exposure levels to CO (carbon monoxide) during WPS may result in a reduction in the amount of oxygen delivered to tissues, including the heart. Furthermore, it is well known that hypoxia is a powerful stimulator of a number of autonomic processes, increasing resting HR, BP, and cardiac output. These results counter the damage reduction claims of purportedly "herbal" waterpipe products and are consistent with non-clinical research employing a waterpipe machine that mimics a human being [49]. Due to a lack of data from long-term trials, it is impossible to say how much WPS may be long-term hemodynamically damaging.

A strong association of WPS with elevated TG, LDL cholesterol, and lower HDL cholesterol levels, all known to be CVD risk factors that encourage atherosclerosis, was found when data from the available studies were combined. As is common knowledge for CS, the underlying mechanisms are not entirely understood. However, it has recently been proposed that TG/HDL imbalances are linked to insulin resistance [50]. Our findings, which demonstrated a significantly higher FBG (fasting blood glucose) in waterpipe smokers compared to non-smokers, are consistent with this. It has been previously documented that CS increases coagulation factors' activity and the risk of thrombosis [51].

Similarly, WPS is associated with higher fibrinogen levels, which may increase thrombogenicity and raise the risk of CV events [35]. Clinical evidence for the probable involvement of WPS in vascular disease can be seen in the elevated CAC (coronary artery calcium) score, and the acute and chronic endothelial dysfunction found among waterpipe smokers [20,28,32,39,41]. Our research on the impact of WPS on the CV system explains and supports findings from studies that found a connection between WPS and CVD incidence, poorer clinical outcomes, and projected prognoses [34,36,41].

The comparison between WPS and CS is crucial because the CV effects of CS are well-known [50]. Unfortunately, there were fewer articles available for this comparison. The main drawback may be the dearth of studies comparing the incidence of CV and cerebrovascular events in waterpipe smokers to those of cigarette smokers. Our findings, however, suggest that the non-acute effects of WPS on the great majority of relevant CV measures are comparable to those brought about by CS. The limited studies did not clearly distinguish between WPS and CS regarding CVD incidence [34,40]. Additionally, compared to cigarette smokers, waterpipe smokers have a higher incidence of CVD complications and mortality [34]. This could be attributed to prolonged WPS, which would result in greater levels of hazardous chemicals breathed and detrimental consequences on the CV system [52]. Our analysis had similar results to a meta-analysis that studied randomized studies as well [53]. Although ours had non-randomized studies, it still proved to be useful in formulating evidence regarding the CV impact of tobacco use. There are several reasons why non-randomized studies, such as observational and cohort studies, can be useful in addition to randomized controlled trials (RCTs) in studying the effects of tobacco use and WPS on CV health. Non-randomized studies can provide valuable information on the patterns of exposure and outcome in real-world populations, which may be different from those in RCTs. This can increase the generalizability of findings to the general population. Non-randomized studies can be useful in assessing the long-term effects of exposure over time, which may not be captured in RCTs that are usually shorter. These types of studies can be useful in identifying populations that may be at particularly high risk of harm from tobacco use and WPS, which can help inform public health interventions. Non-randomized studies can be less expensive and more feasible to conduct than RCTs, which can be logistically and financially challenging [54]. Furthermore, they can be useful in identifying potential mechanisms of harm, which can inform future research and intervention development. Lastly, such studies can generate hypotheses for further investigation in RCTs. It is important to note that both randomized and non-randomized studies have their own limitations and strengths. While RCTs can provide the highest level of evidence for causality, non-randomized studies can provide important information on real-world patterns of exposure and outcomes and can complement the findings from RCTs [54].

On the other hand, the long-term effects of WPS should be considered, as a recent mouse model that revealed cessation of smoking relieves waterpipe smoke-induced hypercoagulability and cardiac injury [55]. It is possible the CV effects resolve in most of the patients with cessation of WPS. Considering the other long-term complications, such as an increase in the incidence of cancer and death attributable to WPS, highlights the pressing need to encourage smoking cessation [56].

Limitations

The comparison of WPS and CS may be the most significant aspect of the review; however, there are not many studies that can be used for this comparison, and the absence of research that reflects the frequency of CV and cerebrovascular events is the biggest drawback. However, some waterpipe users might have previously smoked cigarettes. The amount of time spent smoking cigarettes likely influences the results. Most studies miss this information. As a result, it is impossible to do a meta-regression that takes the period since CS cessation in waterpipe smokers. This is relevant to the other findings that revealed waterpipe users to have a worse cardiometabolic profile than non-smokers, as many studies did not account for all potential confounders when comparing.

## Conclusions

The prevalent misconception that WPS is safe and does not involve smoking still exists. The variety of WPS's short- and long-term CV effects are described in this article. Despite the aforementioned restrictions, the degree of evidence implies that WPS is linked to significant negative effects on the CV system, which resemble those documented for CS. In particular, non-randomized studies are a helpful tool where randomized studies are difficult and still yield results that although may have some bias but enough to draw useful and similar conclusions to randomized trials.
